# Synthesis and physicochemical characterization of carbon quantum dots produced from folic acid

**DOI:** 10.1038/s41598-023-46084-1

**Published:** 2023-10-30

**Authors:** Walaa Fawaz, Jameela Hasian, Ibrahim Alghoraibi

**Affiliations:** 1 Department of Pharmaceutics and Pharmaceutical Technology, Faculty of Pharmacy, Damascus University, Damascus, Syria; 2 Department of Physics, Faculty of Science, Damascus University, Damascus, Syria

**Keywords:** Materials science, Nanoscience and technology

## Abstract

The rising interest in carbon dots (c-dots) is driven by their remarkable potential in the field of biomedical applications. This is due to their distinctive and adjustable photoluminescence characteristics, outstanding physicochemical properties, excellent photostability, and biocompatibility. Herein, carbon dots were successfully produced via the heat synthesis method and characterization for physical and chemical properties using UV–Vis spectrophotometer, photoluminescence spectroscopy, Fourier Transform Infrared and Raman spectroscopy, Energy-dispersive X-ray analysis, and quantum yield. The resulting carbon dots exhibited a distinct blue fluorescence upon exposure to ultraviolet radiation with a 366 nm wavelength. The photoluminescence spectrum of carbon dots displayed a fluorescence peak around 470 nm when excited with a 325 nm wavelength. The synthesized carbon dots demonstrated thermal stability and maintained photoluminescence intensity under different pH conditions, including neutral and alkaline mediums, and good salt resistance ability. Raman spectroscopy confirmed the presence of structural defects within the carbon dots, which are associated with the presence of hybrid groups on their surface. Fourier-transform infrared analysis detected various carbon-bonded, nitrogen-bonded, and oxygen-bonded units. The quantum yield was around 8.9%. These findings from our experiments indicate that the manufactured carbon dots possess substantial promise for a wide range of applications within the biotechnology field.

## Introduction

Fluorescent nanoparticles, including quantum dots, polymer-based fluorescent nanoparticles, and liposome-derived counterparts, have diverse applications in biotechnology, electronics, and sensor fields due to their high quantum yield and vibrant fluorescence properties^1^. Carbon dots have recently gained substantial attention as a fluorescent material, particularly in biological applications such as bioimaging and biosensing. Their appeal lies in their exceptional attributes, including strong fluorescence, aqueous solubility, low toxicity, and biocompatibility^2,3^. Furthermore, carbon dots offer several advantages, such as minimal photobleaching, robust stability, biocompatibility, and low toxicity, making them suitable for a wide range of materials analysis and investigations^4^. Traditional methods for detecting interactions of nanoparticles with target tissues often require specialized, complex equipment and time-intensive procedures. However, nanotechnology offers rapid, straightforward, and cost-effective alternatives by harnessing the photoluminescence property of carbon dots^5^. Carbon dots can be synthesized using two primary approaches: the Top-Down and Bottom-Up methods. In the Top-Down approach, materials like graphene, graphene oxide sheets, carbon nanotubes, carbon fibers, and carbon graphite are fragmented to yield fluorescent carbon structures characterized by *sp*^2^ hybridization. This is typically achieved through laser ablation, arc discharge, or exfoliation methods (e.g., thermal, acid, ultrasonic, or chemical)^6,7^. Acid-oxidizing exfoliation methods rely on acids such as HNO3 and H2SO4 to peel carbonaceous materials into C-dots. However, these acids can adversely affect the original structure of graphitic precursors, incur high purification costs, and entail the use of toxic chemicals^8,9^. Despite the Top-Down method has advantages, including precise control over quantum yield, it requires specialized equipment and, in some instances, hazardous chemicals. The Bottom-Up approach relies on physical or chemical techniques like hydrothermal, microwave-assisted, or thermal pyrolysis. This approach has garnered interest due to its utilization of various precursor molecules, cost-effectiveness, and avoidance of toxic chemicals. Hydrothermal synthesis, characterized by its simplicity, uniform particle dimensions, and high quantum yield, is among the most widely employed methods for carbon dot synthesis. The microwave irradiation method, known for its ease and commercial viability, typically encompasses three steps: polymerization, drying, and carbonization. Various materials such as citric acid, urea, glycerol, amino acids, and polyamines are employed in carbon dots synthesis via water pyrolysis or microwave radiation methods. The optical and fluorescent properties of carbon dots are significantly influenced by the choice of precursors, given their direct impact on carbon content and functional groups. To broaden the scope of carbon dots applications and harness different physicochemical properties, various available materials can be employed in their synthesis, or novel functional groups containing elements like nitrogen, phosphorus, sulfur, and boron can be involved during the synthesis processes. These surface functional groups may facilitate subsequent modifications, influencing particle characteristics, including size, solubility in different solvents, and chemical properties, all of which hinge on the functional groups of the initial materials^10–12^. Folic acid, a compound essential to cellular metabolism, features folate receptors on the surface of various cells, including hepatocytes. Cancer cells also consume folic acid during their division process. By initiating the carbon dot synthesis process with folic acid as a precursor employing temperatures equal to or below 200 °C, the structural memory of folic acid can be preserved. This facilitates specific uptake by cancer cells through folate receptors, thereby advancing therapeutic objectives^13^. Herein, our study aims to synthesize and characterize carbon dots obtained from folic acid that may be used to target drug delivery systems with high specificity for cancer cells.

## Results and discussion

### Synthesis of carbon dots

**Figure 1:**
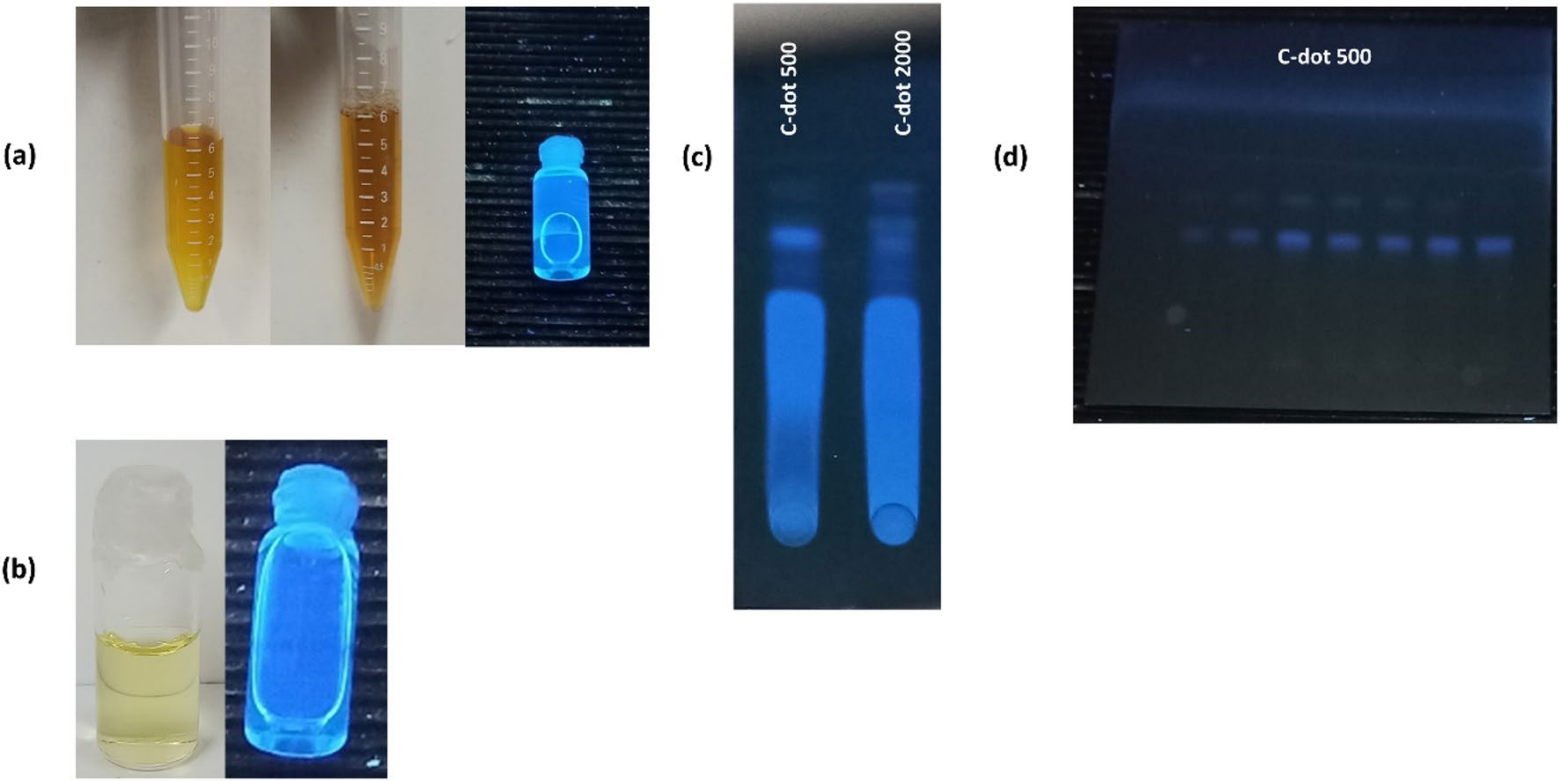
Change of the solution color after preparation of c-dots, c-dots upon exposure to ultraviolet 366 nm (**a**) The prepared carbon dots after four months, under normal light and UV light (366 nm) (**b**) Separation via thin-layer chromatography of the prepared carbon dots (**c**) Thin-layer chromatography for samples with different concentrations of c-dot 500 (**d**).

Carbon dots were synthesized using a heating method at 90 °C to induce the carbonization process. A significant transformation occurred as the solution changed from yellow to a dark brown hue, indicating the successful preparation of carbon dots. Additionally, upon exposure to ultraviolet radiation with a 366 nm wavelength (Fig. 1a), the resulting solution exhibited a distinct blue fluorescence. Furthermore, the carbon dots demonstrated stable fluorescence for several weeks and the samples showed no signs of precipitation or degradation (Fig. 1b).

### The separation via thin-layer chromatography (TLC)

Thin-layer chromatography (TLC) was utilized to investigate the outcomes of the reaction and separation processes of the prepared carbon dots (Fig. 1c). This analysis revealed a distinct fluorescent band for c-dot 500, while multiple bands with varying fluorescence intensities were observed for c-dot 2000. These findings imply that the separation and purification processes of the prepared carbon dots were more efficient when using dialysis bags with pore sizes of 500 Daltons. Additionally, samples containing different concentrations of c-dot 500 (ranging from 2 to 8 μg/ml) were examined. The results showed complete migration of the samples, with a gradient in fluorescence intensity corresponding to the increasing carbon dot concentration (Fig. 1d). The full image of c-dots spots was placed in the supplementary file.

### Spectroscopic scanning using UV–Vis spectrophotometer

**Figure 2:**
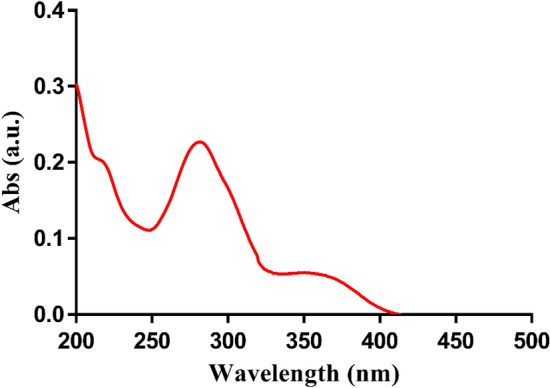
UV–Vis absorption spectra of synthesized carbon dots.

The absorption spectrum of the carbon dots was also examined (Fig. 2). A prominent peak emerged at a wavelength of 280 nm, attributed to the π-π* orbital transition associated with the *sp*^2^ hybridization pattern of the carbon–carbon double bonds (C=C). Furthermore, a shoulder was observed around 360 nm, corresponding to the n-π* orbital transition with the *sp*^3^ hybridization pattern, indicative of bonds between carbon atoms and other functional groups containing nitrogen and oxygen atoms^14^.

### Quantum yield

The quantum yield of c-dot 500 was 8.9%, using quinine sulfate as the reference substance for the calculation. This result aligns with the findings of Bhunia et al.^13^. However, the quantum yield of c-dot 2000 was 3.5%, indicating a clear correlation between the purity of carbon dots and their quantum yield^15^. It is important to note that the quantum yield value is influenced by the surface structure of the carbon dots and potential modifications made during the manufacturing process. Carbon dots derived from common reaction precursors containing electron-withdrawing groups (EWGs), such as carboxyl and epoxy groups, tend to reduce the electron density within the carbon dots, resulting in a relatively lower quantum yield. Typically, carbon dots prepared from materials like graphite or citric acid exhibit a quantum yield that peaks at around 10%^16^.

### Photoluminescence spectroscopy

**Figure 3:**
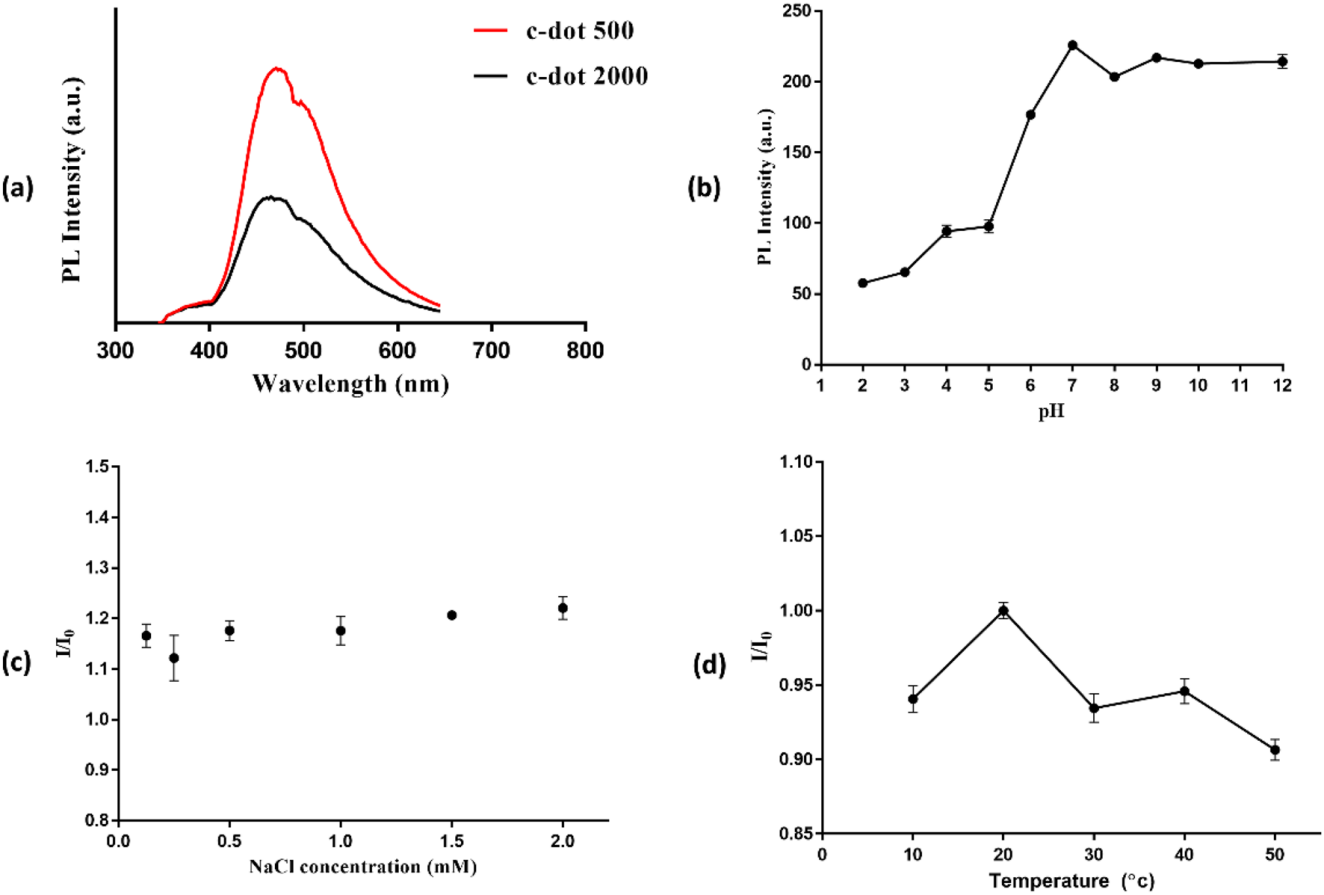
(**a**) The photoluminescence (PL) spectrum of the prepared carbon dots. PL Intensity at different conditions: (**b**) pH variation, (**c**) NaCl concentration, (**d**) temperature.

The carbon dots photoluminescence (PL) spectrum revealed a prominent fluorescence peak in the blue region, specifically around 470 nm when excited using a 325 nm helium-cadmium laser (Fig. 3a). Notably, c-dot 500 exhibited higher PL intensity compared to c-dot 2000. The photoluminescence intensity is directly associated with the excited absorption band, showing higher emission intensity when the n-π* band is stimulated. Conversely, lower intensity occurs when excitation occurs within the far-ultraviolet range, particularly within the π-π* absorption band. In this context, the appearance of photoluminescence peaks depends on the structural composition of the carbon dots and the presence of various elements on their surface^9^. On the other hand, to assess the anti-interference capability of carbon dots' fluorescence, PL intensity was examined under different pH levels, temperature conditions, and in the presence of different concentrations of sodium chloride solution. The PL intensity exhibited variability in response to changes in the pH of the medium (Fig. 3b). The PL intensity decreased in acidic conditions, reached the maximum peak at a neutral pH, and subsequently decreased again before stabilizing in the basic medium. In contrast, (Fig. 3c) shows no significant alteration of PL intensity at different NaCl concentrations (0.1–2 mM). Additionally, the carbon dots demonstrated remarkable stability, retaining 90% of their PL intensity when subjected to a temperature of 50 °C, as shown in (Fig. 3d).

### Raman spectroscopy

**Figure 4:**
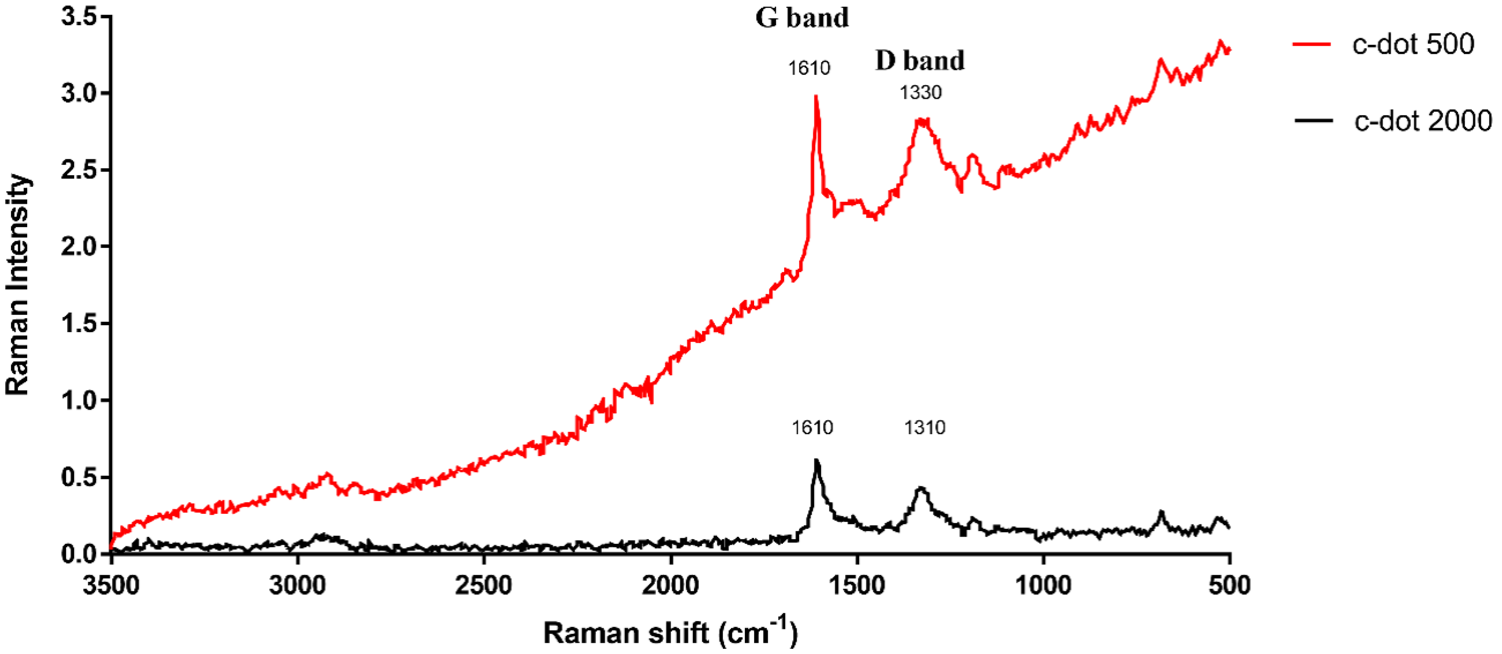
Raman spectra of synthesized carbon dots (excitation wavelength 785 nm).

Raman spectroscopy is instrumental in elucidating structural characteristics associated with defects and irregularities in graphene-based materials. The Raman spectra (Fig. 4) exhibit a peak at 1610 cm^−1^, identified as the “Graphene band.” This peak signifies the presence of *sp*^2^ hybridization pattern, characteristic of the hexagonal arrangement of carbon atoms, serving as a distinct hallmark of materials with a regular crystalline structure. Concomitantly, another peak emerges at 1330 cm^−1^, denoted as the “Defect band.” This feature indicated the presence of disorder and defects within the carbon material, often arising from carbon atoms displaying an *sp*^3^ hybridization pattern. To quantify the extent of disorder and defects within the graphene structure, the *I*_D_/*I*_G_ ratio is used to quantify the extent of disorder and defects within the graphene structure. There is a regime of “low” defect density where *I*_D_/*I*_G_ will increase as a higher defect density creates more elastic scattering^17^. For c-dot 2000 and c-dot 500, the measured *I*_D_/*I*_G_ ratios were 0.69 and 0.94, respectively. This observation validates the presence of defects within the carbon dot structure, which influence the *sp*^2^ hybridization pattern in the carbon structure and indicate the presence of hybrid groups on the surface^18^. Notably, these *I*_D_/*I*_G_ ratios fall within the reported range for high-quality carbon dots^19^.

### Fourier transform infrared spectroscopy

**Figure 5:**
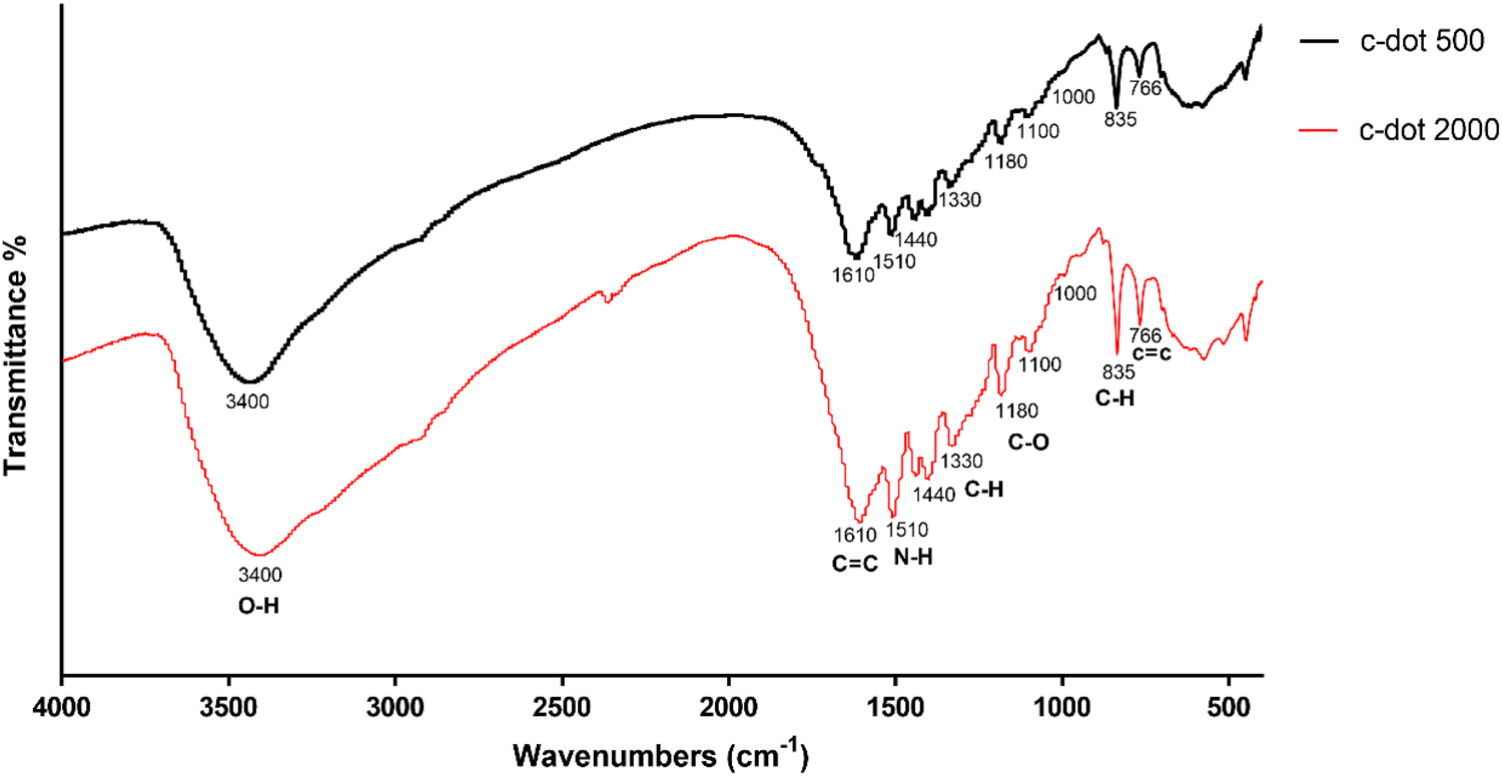
FTIR spectra of synthesized carbon dots.

FTIR spectroscopy reveals the presence of various functional groups within the samples. In the infrared spectra of c-dot 500 and c-dot 2000, distinct absorption peaks at specific wavenumbers were observed **(**Fig. 5**)**. The broad absorption peak observed at 3440 cm^−1^ is attributed to the presence of OH groups, which are associated with water molecules absorbed within the carbon dots sample. At 1610 cm^−1^, an absorption peak emerges, indicating the stretching of the C=C bond and signifying the stability of the graphitic structure. Furthermore, the peak at 1510 cm^–1^ signifies the presence of the N–H bond, characteristic of the amine group. At 1330 cm^–1^, the absorption peak corresponds to the C–H bond. Another noteworthy observation is the absorption peak at 1440 cm^–1^, suggesting the existence of the carboxyl group attributed to the vibration of the C–O bond within this functional group. Additionally, the peak at 1180 cm^−1^ is associated with the stretching of the C–O band. A smaller, shoulder-like peak appearing around 1000 cm^−1^ signifies the vibration of the C–O bond in the epoxy groups (C–O–C). The presence of the peak at 835 cm^−1^ corresponds to the vibration of the C–H bond, while the peak at 766 cm^−1^ arises due to the stretching of the C=C bond. This particular peak can be attributed to π-π* transitions^20,21^.

### Energy-dispersive X-ray analysis (EDX)

**Figure 6:**
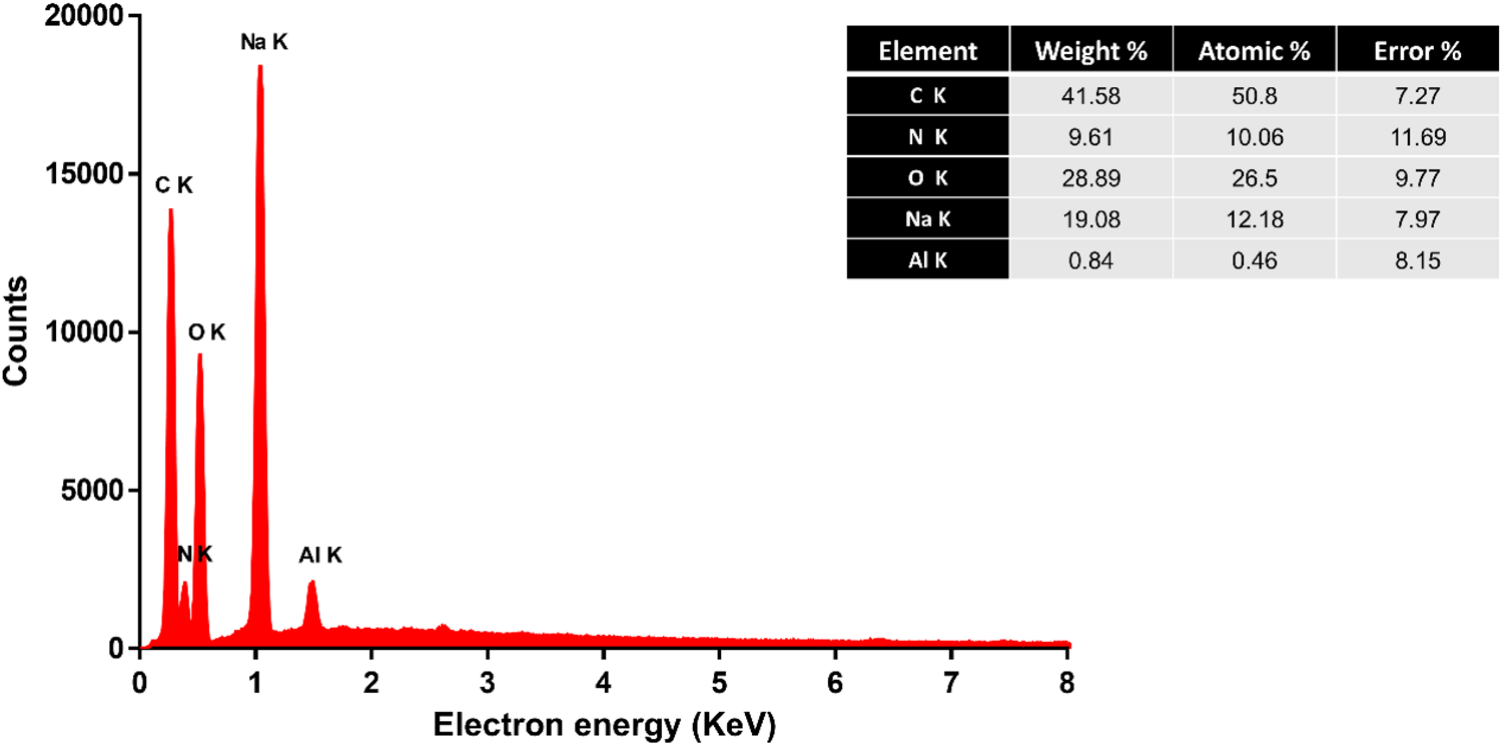
Energy-dispersive X-ray analysis of prepared carbon dots.

The EDX technique serves as a valuable tool for scrutinizing the elemental composition of carbon dots, providing insights into their purity^11^. The analysis was employed to determine the elemental composition of the carbon dots and subsequently estimated the weight and atomic ratios of carbon, oxygen, and nitrogen based on the EDX data (Fig. 6). The sample indicated atomic percentages of 50.8% for carbon, 9.61% for nitrogen, and 28.89% for oxygen. Additionally, the analysis revealed the presence of 19% sodium within the sample. This sodium presence can be attributed to the utilization of sodium hydroxide during the carbon dot synthesis process.

## Conclusion

Carbon dots were effectively synthesized via the heating method. In terms of purification, it was observed that using dialysis bags with 500 Dalton pore sizes yielded superior results when compared to dialysis bags with 2000 Dalton pore sizes. This was evidenced by the separation process observed through thin-layer chromatography. The purity of the prepared sample had a notable impact on both quantum yield and photoluminescence intensity, with c-dot 500 exhibiting higher values. The carbon dots' photoluminescence (PL) spectrum exhibited a notable fluorescence peak at approximately 470 nm when excited with a 325 nm wavelength. The synthesized carbon dots exhibited good thermal stability and retained high photoluminescence intensity when subjected to changing pH conditions, including neutral and alkaline mediums, as well as in the presence of different concentrations of NaCl solution. Raman spectroscopy confirms the presence of defects within the carbon dot structure belonging to the existence of hybrid groups on the surface of the c-dots. The Fourier-transform infrared analysis revealed the presence of various carbon-bonded, nitrogen-bonded, and oxygen-bonded units originating from the synthesis process that involved folic acid as the reagent for C-dots preparation. Based on our experimental findings, the synthesized carbon dots show promise for extensive applications in biotechnology.

## Methods

### Synthesis of carbon dots

Carbon dots were prepared according to the method outlined by Bhunia et al.^13^ with minor adjustments. Initially, 5 mL of deionized water was combined with 100 mg of folic acid, followed by the addition of 400 μL of a 20 N sodium hydroxide solution to the folic acid solution. This mixture was then heated to 90 °C for a duration of 4 h. To effectively separate the carbon dots from any unreacted by-products, a dialysis approach was employed. For optimization of the separation process, dialysis bags with pore sizes of 500 and 2000 Daltons were employed, and the dialysis was carried out against 1 L of deionized water for a period of 24 h The carbon dots that were prepared were labeled based on the dialysis bag utilized as c-dot 500 and c-dot 2000.

### Carbon dots characterization

#### The separation via thin-layer chromatography (TLC)

Carbon dots were applied as a 20 μL spot onto the bottom of the silica gel-coated aluminum TLC plate (TLC silica gel 60 F254, Sigma-Aldrich, Canada). The plate was developed in [ethyl acetate (60), methanol (30), deionized water (15), and formic acid (1)] (V/V). The fluorescence of the samples was detected via UV light at 366 nm with a GAMAG UV-light cabinet.

#### Spectroscopic scanning using UV–Vis spectrophotometer

The absorption spectrum of the prepared carbon dots was systematically examined within the wavelength range spanning from 200 to 500 nm. UV–Vis spectroscopy (6850 UV/Vis. Spectrophotometer-JENWAY) was employed to assess the optical characteristics of the carbon dots, given their propensity for strong UV absorption. This spectroscopic technique operates on the fundamental principle of the interaction between chemical compounds and ultraviolet or visible light. As light is absorbed by the material, it undergoes excitation and subsequent de-excitation processes, resulting in the generation of distinct and characteristic spectra^11^.

#### Photoluminescence spectroscopy

The samples were excited by a helium-cadmium laser emitting a 325 nm wavelength beam, followed by the quantification of photoluminescence intensity through spectrum analyzer (monochromator, jobin Yvon, France).

### Quantum yield

The quantum yield was calculated using quinine sulfate as the standard (QY = 54%) according to the following equation:

$${\left(QY\right)}_{sm}= {\left(QY\right)}_{st}\frac{{(PL}_{area}{\div OD)}_{sm}}{{(PL}_{area}{\div OD)}_{st }}* \frac{{{\eta }^{2}}_{sm}}{{{\eta }^{2}}_{st}}$$

The quantum yield of carbon dots indicated as (QY)sm, is determined in relation to the quantum yield of the standard (quinine sulfate), represented as (QY)st. PLarea refers to the fluorescence area, and η represents the refractive index of the solvent, which is 1.33 for water.

### Fourier transform infrared spectroscopy

The composition of functional groups on the surface of carbon dots typically varies based on the preparation method and materials employed. As such, FTIR technology is utilized to identify and characterize these surface functional groups.

### Raman spectroscopy

The Raman spectrum is a valuable tool for studying the vibrational patterns within a substance, providing valuable insights into its molecular structure, crystallinity, and various structural characteristics. In this study, the Raman spectrum was obtained using an excitation wavelength of 785 nm for the synthesized carbon dots.

### Energy-dispersive X-ray analysis (EDX)

Energy dispersive X-ray (EDX) is a method employed to quantitatively analyze elements with distinct X-ray spectral signatures. It offers comprehensive data regarding the elements within the sample, including both their atomic and weight ratios**.**

### Supplementary Information

Supplementary Information.

## Data Availability

Correspondence and requests for materials should be addressed to W.F.
