# Supplementary material for: Synthesis and physicochemical characterization of carbon quantum dots produced from folic acid
Source: Sci Rep. 2023 Oct 30;13:18641. doi: 10.1038/s41598-023-46084-1 (PMC10616078; doi:10.1038/s41598-023-46084-1)
Supplement: Supplementary file 1 — Supplementary Information. [file 41598_2023_46084_MOESM1_ESM.docx]

**Synthesis and Physicochemical Characterization of Carbon Quantum Dots Produced from Folic Acid**

**Walaa Fawaz^1*^, Jameela Hasian^1^, Ibrahim Alghoraibi^2^**

**^1^Department of Pharmaceutics and Pharmaceutical Technology, Faculty of Pharmacy, Damascus University, Damascus, Syria.**

**^2^Department of Physics, Faculty of Science, Damascus University, Damascus, Syria.**

*Address correspondence to this author at the Department of Pharmaceutics and Pharmaceutical Technology, Faculty of Pharmacy, Damascus University, Damascus, Syria; Tel/Fax: 00 963 11 2131871 , 00 963 11 2119837; E-mails: fwalaa33@gmail.com , walaa.fawaz@damascusuniversity.edu.sy.


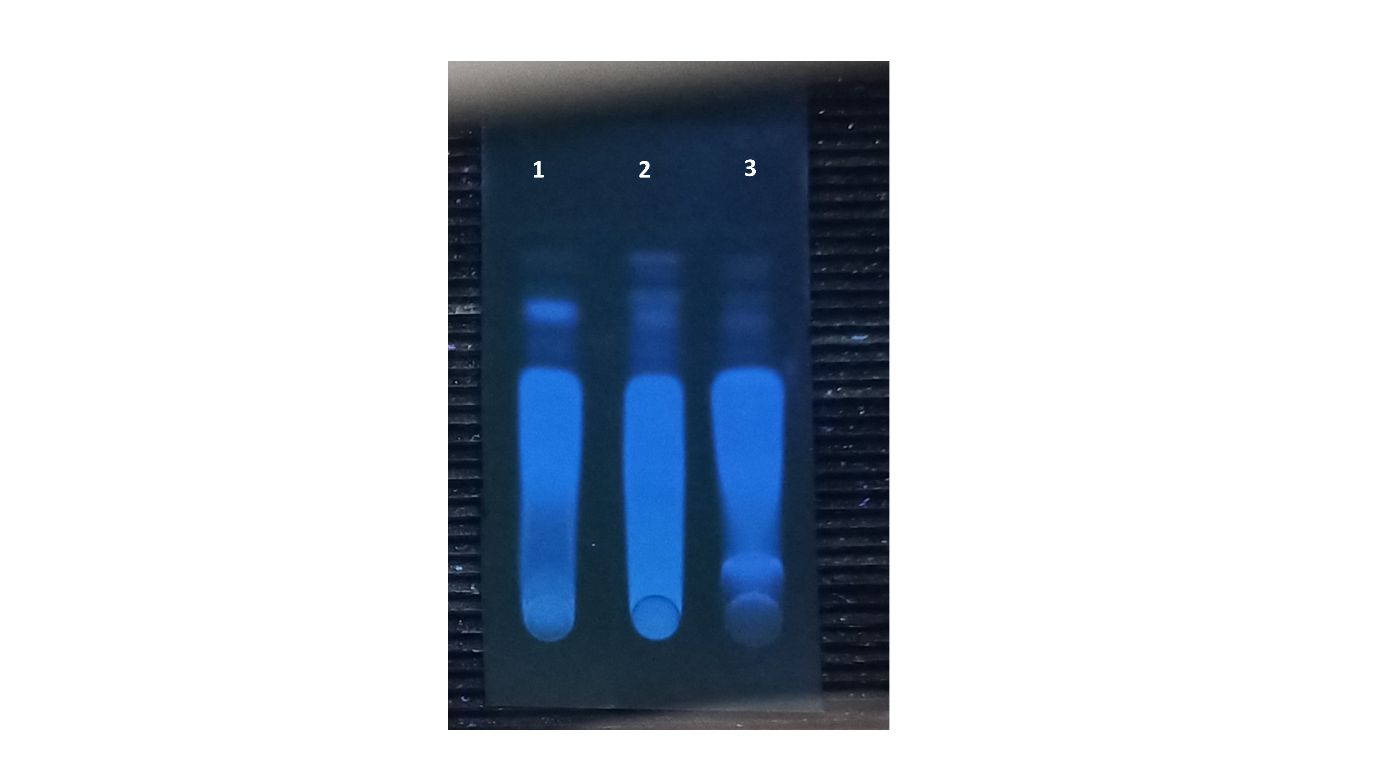


**Figure (1S).** Separation via thin-layer chromatography of the prepared carbon dots. **(1)** c-dot 500. **(2)** c-dot 2000. **(3)** An additional sample whose result was not included in the manuscript.
